# Usefulness and misrepresentation of phone surveys on COVID-19 and food security in Africa

**DOI:** 10.1007/s12571-022-01330-8

**Published:** 2022-12-14

**Authors:** Tilman Brück, Mekdim D. Regassa

**Affiliations:** 1 Economic Development and Food Security, Leibniz Institute of Vegetable and Ornamental Crops (IGZ), Großbeeren, Germany; 2 Faculty of Life Sciences, Humboldt-University of Berlin, Berlin, Germany; 3 ISDC - International Security and Development Center, Berlin, Germany

**Keywords:** Phone survey, COVID-19, Food security, Africa, I1, I3, C83

## Abstract

We survey efforts that track food security in Africa using phone surveys during the COVID-19 pandemic. Phone surveys are concentrated in a few countries mostly focusing on a narrow theme. Only a few allow heterogeneous analyses across socioeconomic, spatial, and intertemporal dimensions across countries, leaving important issues inadequately enumerated. We recommend that the scientific community focuses on countries (and regions and groups within countries) where the evidence base is thin, and that policymakers in less researched areas attract more research by improving their statistical capacity, openness, and governance.

## Introduction

The COVID-19 pandemic and its countermeasures have shaped lives and livelihoods around the world, causing economic contractions (IFPRI, 2020), worsening poverty (Laborde et al., 2021) and food insecurity (Dasgupta & Robinson, 2022; Egger et al., 2021; Jaacks et al., 2021). Given their weak economic and health care systems and largely immunocompromised populations, African countries carry a particularly heavy burden in terms of COVID-19 induced welfare losses (Djankov & Panizza, 2020; IFPRI et al., 2020). At the same time, COVID-19 risks reinforcing pre-existing socioeconomic disparities within and across countries in the region (Nechifor et al., 2021; Poudel & Gopinath, 2021).

Empirical evidence on the scale and the nature of the impacts of the pandemic and its countermeasures, while growing, is quite limited, partly due to the lack of suitable, comparable, and timely micro-level data (Delius et al., 2020; Gourlay et al., 2021). This lack of data also stems from the way COVID-19 is transmitted person-to-person, which inhibits face-to-face survey data collection. To overcome this challenge, high-income countries have managed to rely on real-time economic data as well as web-based surveys. In low-income countries in Africa, these options were not widely available and may even have worsened as the National Statistical Offices (NSOs) in these countries were hit particularly hard by the pandemic (UNDESA and World Bank, 2020)^1^. Fortunately, the nascent expansion of mobile phone subscriptions as well as the learning experience from the 2014 Ebola outbreak in West Africa and the 2017 drought- and conflict- related food insecurity crisis in West and East Africa helped to deploy phone surveys quickly at the beginning of the ongoing pandemic (Gourlay et al., 2021; Hoogeveen & Pape, 2020).

The application of the phone surveys involves several challenges. First, phone surveys involve constraints regarding the type and size of questions that could be included in the interviews. In order to limit respondent fatigue, interview questions need to be kept short and simplified and answer choices limited (e.g., yes or no). Furthermore, interlinked and complex questions such as consumption modules are difficult to include in phone surveys (Hirvonen et al., 2021). While such a concern about respondent fatigue is not uncommon in surveys in general (Ambler et al., 2021), it is more pronounced in phone surveys (Abate et al., 2021). Second, contrary to face-to-face surveys, phone surveys don’t allow enumerators to observe visual non-verbal cues from respondents. While rigorous training of enumerators and certain lead-in scripts and probes could help identify and reduce the problem, they don’t fully address it (Dillon, 2012). Relatedly, the absence of in-person communication during phone interviews might make it difficult to build trust with the respondents, introducing willful error by a respondent, especially if the questions are sensitive (Dabalen et al., 2016).

There are also certain limitations that are more relevant with respect to conducting phone surveys in Africa and low-income countries’ settings in general. First, sampling bias is a concern since the survey could only be administered to respondents with working phones and phone ownership varies systematically across and within countries based on sociodemographic characteristics (e.g. age, education and wealth status) and place of residence (rural vs. urban areas) (Dabalen et al., 2016; Dillon, 2012; Kühne et al., 2020). While the use of representative baseline survey data could reduce the bias, it does not fully eliminate it (Ambel et al., 2021). Another limitation of phone surveys in Africa relates to the availability and the systematic variation in infrastructure particularly electricity and mobile signal, which is rampant in the continent (World Bank, 2009). Such a disparity among locations effectively creates a sampling problem by introducing bias, since availability and quality of infrastructure is likely to be correlated with other important characteristics, such as urban proximity, availability and quality of public goods (e.g. road, health centers, water supply) and average wealth (Dillon, 2012).

Notwithstanding their shortcomings, phone surveys have proven useful and cost-effective in collecting data in remote and conflict areas and in circumstances where face-to-face data collection appears to be risky to the safety of the enumerators and the survey respondents (Dabalen et al., 2016; Delius et al., 2020; Hoogeveen & Pape, 2020; Sturges & Hanrahan, 2004). Due to this and following the onset of the ongoing pandemic, large number of phone-based interviews are being conducted throughout the continent. However, we currently lack an overview of efforts to trace food security in Africa using phone surveys, risking duplication or omission of data collection efforts. We address this knowledge gap by reviewing all phone surveys tracking food security in Africa since the beginning of the pandemic, including our own phone survey called Life with Corona-Africa (LwC-Africa). We concentrate our review on five key issues, namely the topical, temporal, and geographic dimensions as well as geospatial coding and open access of the data.

It is true that phone surveys came to prominence in Africa due to the COVID-19 pandemic. However, their use might continue into the future as a standalone data collection model or in combination with face-to-face interviewing (Gourlay et al., 2021). The experience during the pandemic highlights that data such as those obtained through phone surveys have the potential to strengthen and modernize core data collection programs and be a key component of the national data systems (UNDESA and World Bank, 2020). Therefore, our review is helpful to highlight the broader picture of the size, the content, and the spatial and temporal distribution of the phone surveys as well as identify evidence gaps to inform future designs.

The rest of the paper is organized as follows. The next section first presents the data sources used in the paper and then describes the timeline, distribution and contents of the phone surveys. Section 3 discusses the implication of the results. Section 4 concludes.

## Data and results

### Data sources

For our review, we searched for phone-based surveys on COVID-19 and food security in Africa since the beginning of the pandemic in four steps^2^. First, we searched international repositories for registered COVID-19 and food security-related surveys and projects on Africa: the central registry of American Economic Association; the Economics Observatory (ECO) of European Economic Association (EEA), and the RECOVR research hub of Innovation for Poverty Action (IPA). Second, we searched for mentions of phone surveys in blogs, news articles, policy briefs, and academic literature on the websites of Google Scholar, IPA, and Relief Web, combining the terms (“COVID 19” OR “COVID-19” OR CORONA OR coronavirus), “food security”, (“phone survey” or “telephone survey”) for Africa, for the sub-regions and the individual countries. Third, and building on the findings of step two, we searched on the websites of African national statistical offices and several international organizations (World Bank, FAO, WHO, and WFP) using the same search terms. Finally, we evaluated all identified phone surveys to compile our final census of phone-based surveys on COVID-19 and food security in Africa since the beginning of the pandemic.

To examine the correlates of phone survey intensity, we used several indicators extracted from multiple data sources including the World Development Indicators (WDI) at https://data.worldbank.org/indicator, Fragile State Index (FSI) generated and made available by the Fund for Peace (FFP) at https://fragilestatesindex.org/ and COVID-19 caseloads and deaths from https://ourworldindata.org.

### Results

#### Description of phone surveys

Our search yielded 234 completed or ongoing phone surveys on COVID-19 in Africa as of November 15, 2021 (Table 1)^3^. A large share of these (90, or 39%) are rapid surveillance surveys aimed at assessing knowledge and perceptions of coronavirus. Typically, these are cross-sectional and individual-level opinion surveys conducted at the onset of the pandemic across multiple countries in or including Africa. The major leading organizations of such surveys include Partnership for Evidence-based COVID-19 Response (PERC), 60 Decibels, GeoPoll, and FinMark Trust (Table 5 in the Appendix). The World Bank and WFP are two prominent organizations that have been collecting near real-time phone survey data across most of the countries in the continent. The World Bank capitalized on its pre-pandemic cooperation with national statistical offices (NSO) to collect High Frequency Phone Surveys (HFPS) or Household Monitoring Surveys (HMS) in a large number of countries, including most countries in Africa, to inform a wide range of knowledge products (Gourlay et al., 2021)^4^. To supplement inputs used in its global hunger monitoring system, the World Food Program (WFP) conducts continuous phone-based food security monitoring through call centers. At the end of 2021, the system was already set up in several developing countries, including 26 African countries, to collect data on a rolling basis over a three-month period^5^. Other surveys include rural household surveys implemented by CGIAR Research Centers (e.g. IFPRI), surveys conducted by academic intuitions (e.g., the university of Oxford, and ETH Zürich), and others (e.g., IGZ/ISDC, the hosts of our study, LwC-Africa). Table 9 in the Appendix presents the complete list of all the phone surveys including start time, sample size, number of survey rounds and internet links.

**Table 1:** Major types of phone surveys

Survey types	Number	% share
Rapid surveillance surveys	90	38.6
World Bank-High Frequency Phone Survey (HFPS)	20	8.6
World Bank-Household Monitoring Survey (HMS)	14	6.0
World Food Program-Hunger & COVID monitoring surveys	26	11.2
Rural HH surveys	19	8.2
University led surveys	46	19.7
Other surveys	18	7.7
Total	234	100

Source: Computed from data compiled by the authors

**Fig. 1:**
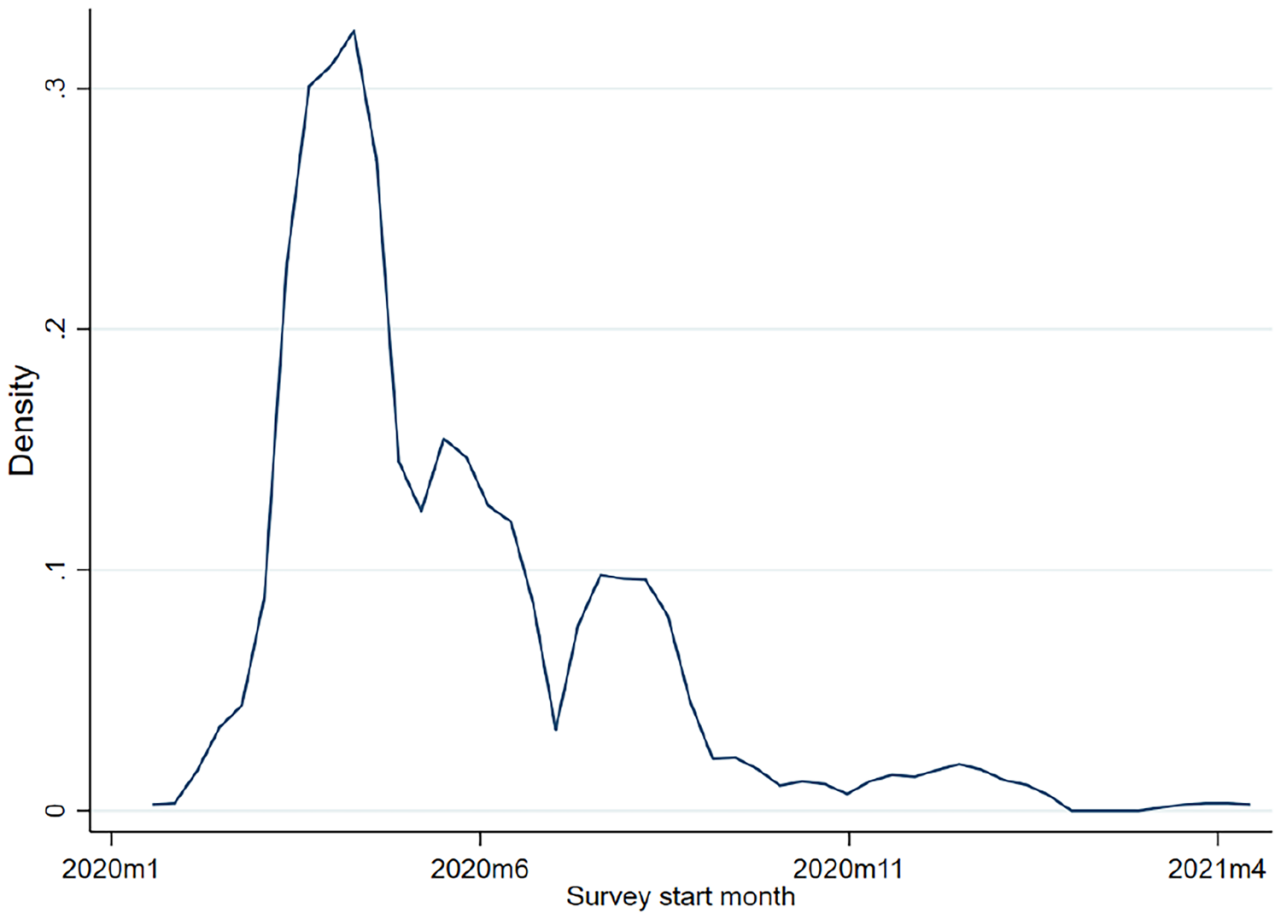
Distribution of phone surveys by starting time (month). Source: Computed from data compiled by the authors

Panel A of Table 2 presents the main descriptive characteristics of the phone surveys. Typically, the surveys we identified are medium-sized (~ 1,000 respondents), cover both rural and urban areas (71%), run for 2 or 3 rounds, collect data for a short period of time (six months or less), focusing on a narrow theme. For a little more than half of the surveys, the unit of analysis are individuals (57%) and the same respondents were contacted repeatedly over time (i.e. panel) (53%). In the review, we included our own study, life with Corona Africa (LwC-A), collecting phone surveys in four African countries — Uganda, Tanzania, Sierra Leone, and Mozambique (see Table 6 in the Appendix for brief description of the survey). About three-quarters of the surveys had been started during the first four months of the beginning of the pandemic between March and June 2020. Over the subsequent periods, the number of ongoing surveys tailed off as existing surveys were phased out and few new surveys were started (Fig. 1).

**Table 2:** Description of the phone surveys and the baseline data

Panel A: Description of Phone surveys			
Number of surveys			234
Median number of respondents per survey			1,001
Average number of rounds per survey			2.45
survey covers a few thematic areas, yes = 1			0.55
Survey is panel, yes = 1			0.53
Unit of Analysis			
		Household, yes = 1	0.42
		Individuals, yes = 1	0.57
		Others, yes = 1	0.01
Geographical coverage of surveys			
		Urban areas only, yes = 1	0.15
		Rural area only, yes = 1	0.13
		Both urban and rural, yes = 1	0.71
Survey available for public use, yes = 1			0.24
Duration of surveys			
		6 months or less	0.63
		6–12 months	0.25
		more than a year	0.12

Panel B: Description of baseline data used in phone surveys			
Pre-COVID-19 baseline used, yes = 1			0.38
Type of baseline used			
One-off specialized surveys, yes = 1			0.32
Long running panel surveys			
		Specialized panel surveys, yes = 1	0.27
		Integrated household panel surveys, yes = 1	0.41
If no baseline data, underlying sampling frame			
		Administrative data, yes = 1	0.43
		Random Digit Dial (RDD), yes = 1	0.57
Is the underlying sampling frame nationally representative, yes = 1			0.29

Source: Computed from data compiled by the authors

Panel B of Table 2 presents a description of baseline data used for COVID-19 related phone surveys in Africa. Three major types of baseline survey data are distinguishable. The first type is one-off specialized surveys, typically undertaken before the pandemic for other studies (e.g. rural household surveys conducted by IFPRI) and later adapted to assess the impact of the pandemic. The second type is long-term specialized panel surveys. These are long-running panel surveys specialized by their thematic area or spatial focuses (e.g. the Feed the Future (FtF); the Productive Safety Net Program (PSNP); the Young Lives surveys; Integrated Household Budget Survey). The third type is general-themed, long-running, and integrated household panel surveys. Living Standard Measurement Studies (LSMS); Household Integrated Panel Surveys (HIPS); Life Panel Surveys and the National Income Dynamics Study (NIDS) are the most popular in this category.

When relevant baseline data is not available, two other types of sampling frames are commonly used for phone surveys in lower-income settings. The first is the use of lists of phone numbers, for example from a mobile network operator or contact details of beneficiaries of a program. Another option is to use phone numbers created through random digit dialing (RDD). These two methods jointly account for about 60% of the sampling frame used in phone surveys. Between the two, RDD is slightly more used (Table 2, Panel B).

#### Review of phone surveys

In this part, we concentrate our review on five key dimensions, namely the topical, temporal, and geographic dimensions as well as geospatial coding and open access of the data. These are dimensions that a survey should accommodate to adequately inform the pattern in the evolution and the socio-economic impacts of and responses to the pandemic (Gourlay et al., 2021; Kühne et al., 2020; Stojetz et al., 2022).

(A) Topical dimension

The identified phone surveys vary widely in terms of the topical areas covered. Table 3 shows that survey modules related to COVID-19 exposure and food (in)security are the two most common ones. COVID-19 exposure is typically assessed based on simple yes/no answers to such questions as “whether the respondents think they or somebody they know had COVID-19”. Another related module common among the phone surveys is on adherence to public health and social measures (PHSMs). Many of these involve data collections that elicit information on how much respondents followed hygiene and social distance measures such as hand washing, avoiding large gatherings and wearing face masks.

**Table 3:** Contents of phone surveys

Survey includes COVID exposure indicator, yes = 1	0.97
Survey includes Public Health & Social distancing measures, yes = 1	0.71
Survey allows merging with external data, yes = 1	0.39
Survey includes food security indicator, yes = 1	0.99
Food security standardized, yes = 1	0.66
**Included food security measures**	
Food Consumption Score (FCS), yes = 1	0.18
Food gap, yes = 1	0.60
Food Insecurity Experience Scale (FIES), yes = 1	0.28
Access to food, yes = 1	0.97
Multiple Food security measures included, yes = 1	0.34
**Other welfare measures included**	
Employment status, yes = 1	0.66
Income change, yes = 1	0.79
Access to services, yes = 1	0.59
Mental health, yes = 1	0.22
Coping mechanisms, yes = 1	0.74
Survey includes all the above welfare measures, yes = 1	0.06
Survey includes half or less of the welfare measures, yes = 1	0.45
Observations	234

Source: Computed from data compiled by the authors

**Table 4:** The distribution of phone surveys across countries

	Surveys		Surveys rounds		Respondents		Interviews	
Countries	Number	Share (%)	Number	Share (%)	Number	Share (%)	Number	Share (%)
Kenya	27	11.5	68	11.9	61,622	15.1	121,247	15.3
Uganda	20	8.5	49	8.6	41,146	10.1	67,078	8.5
Ghana	17	7.3	30	5.2	28,200	6.9	36,901	4.6
Ethiopia	12	5.1	41	7.2	18,750	4.6	91,432	11.5
South Africa	8	3.4	19	3.3	20,388	5.0	61,783	7.8
Nigeria	12	5.1	37	6.5	23,887	5.9	47,181	5.9
Malawi	7	3.0	24	4.2	7,937	1.9	29,841	3.8
Tanzania	9	3.8	22	3.8	13,075	3.2	13,279	1.7
Zambia	10	4.3	20	3.5	16,598	4.1	19,281	2.4
Others	112	47.9	263	45.9	175,974	43.2	305,706	38.5
Total	234	100	573	100	407,577	100	793,729	100

Source: Computed from data compiled by the authors

From among the 234 phone surveys included in the review, 231 (or 98.7%) of them include some indicators of food security, such as changes in income or access to the food market due to the pandemic.^6^ While most of these questions provide useful insight, not all of them reflect real changes in food security (Cafiero et al., 2018). Table 3 shows that only 66% of all surveys contain standardized modules on food security such as the Food Insecurity Experience Scale (FIES), Food Consumption Score (FCS), or the number of months of food shortage (food gap). Access to food, or the lack thereof, is the most commonly used food security module (97%) followed by food gap (60%). FCS and FIES are less frequently used perhaps because they require adding a relatively large number of questions (cf. FCS) or they involve questions that are less straightforward or require extensive enumerator training or monitoring (cf. FIES). The number of surveys that include multiple food security measures is even lower (34%).

Other commonly surveyed welfare measures include changes in employment status (66%), income changes (79%), access to services such as drinking water and health services (59%) and coping mechanisms (74%). Less than a quarter of surveys included mental health questions. This is despite the significant increase in mental health issues since the onset of the pandemic (Abreu et al., 2021; Brülhart et al., 2021). Furthermore, Table 3 shows that the surveys are limited in terms of comprehensiveness. While a clearer understanding of the pandemic requires survey data that cover multiple welfare and behavioral dimensions, more than half of the phone surveys we reviewed mostly focus on a narrow theme (e.g. only one dimension of food security or only the health impact of COVID-19 exposure).

(B) Temporal dimension

Given its comprehensive nature, the full impact of the pandemic might not be apparent in the short term based on one-shot surveys (IFPRI, 2020). To be more useful for research, phone surveys need to be collected throughout the pandemic, covering periods of lockdowns and infection peaks and allow comparison before, during and after. However, Table 2 indicates that about half of the phone surveys are cross-sectional and hence are less useful to assess the evolution, the responses to, and the socioeconomic impacts of the pandemic over time. Even when the surveys are repeated, they typically do not last more than 3 rounds. The average number of survey rounds is 2.5. For about 63% of the surveys, the duration of the surveys – the number of months between the start and end of the survey – is less than 6 months. Only 12% of the surveys cover more than a year (Table 2).

**Fig. 2:**
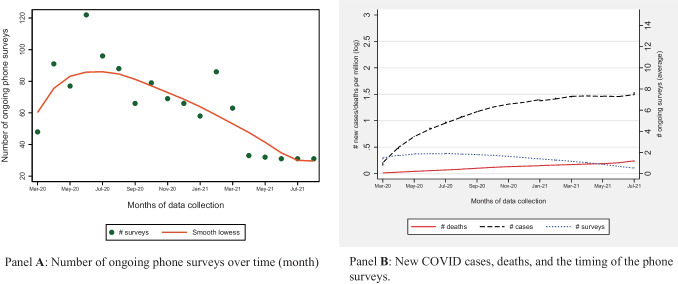
Patterns in new COVID cases, deaths, and number of ongoing phone surveys over time. Sources: Data on new COVID cases and deaths are extracted from ourworldindata.org; the total and average number of ongoing phone surveys is computed from data compiled by the authors

Furthermore, Panel A of Fig. 2 shows that the number of ongoing surveys has continuously been declining. Between March and June 2020, the number of surveys was growing and in June 2020, 120 different surveys were in progress in the continent. Since June, the size has been declining persistently to reach about 30 by the end of the year. Panel B further shows that the number of ongoing surveys has continuously been declining regardless of the progression of the pandemic.

(III) Geographic dimension

The COVID-19 pandemic and the subsequent lockdowns and social distancing measures have largely halted in-person surveys. As a result, following the onset of the pandemic, phone-based surveys became the main, often the only, alternative source of data in most countries in Africa (Gourlay et al., 2021). Given the uncertainty that accompanied the pandemic, phone surveys appeared critical to fully understand, manage and mitigate the human, social and economic effects of the shock. However, the distribution of the phone surveys is highly uneven. Kenya is the most surveyed country in the continent accounting for 11.5% of all phone surveys, amounting to more than 15.3% of all interviews (Table 4). The top five surveyed countries — Kenya, Ghana, Uganda, Ethiopia, and South Africa — account for more than 35% of all surveys and more than 40% of interviews, while accounting for only about 20% of the continent’s population. Other frequently surveyed countries include Nigeria, Malawi, Zambia, and Tanzania. These nine countries account for more than half of the phone surveys related to COVID-19 and food security. The picture remains the same regardless of the measure of survey intensity used – number of surveys, survey rounds, number of respondents, or number of interviews conducted (Table 4).

What may explain the uneven distribution of phone surveys across African countries? To answer this, we investigated the simple bivariate correlations between survey intensity and factors that are broad indicators of the perceived costs or ease of conducting research. These factors include population size, statistical capacity score (SCS), official development assistance (ODA), Fragile State Index (FSI), COVID-19 caseloads and deaths, and the use of English as an official language. We identified these factors from previous literature that looked at the distribution of research across African countries and beyond (Das et al., 2013; Porteous, 2020; Robinson et al., 2006).

We derived data on these indicators from multiple sources. Data on population size, SCS, and ODA are extracted from the World Development Indicators at https://data.worldbank.org/indicator. SCS is a composite score on a scale of 0-100 assessing the capacity of a country’s statistical system on methodology, data sources, and periodicity and timeliness^7^. ODA consists of disbursements of loans made on concessional terms and grants by official agencies of the members of the Development Assistance Committee (DAC), multilateral institutions, and non-DAC countries. FSI is generated and made available by the Fund for Peace (FFP). It summarizes the economic and political instability of countries based on 12 conflict risk indicators^8^. COVID-19 caseloads and deaths refer to the number of COVID-related cases/deaths corresponding to the first three months of the pandemic, and are extracted from: https://ourworldindata.org.

**Fig. 3:**
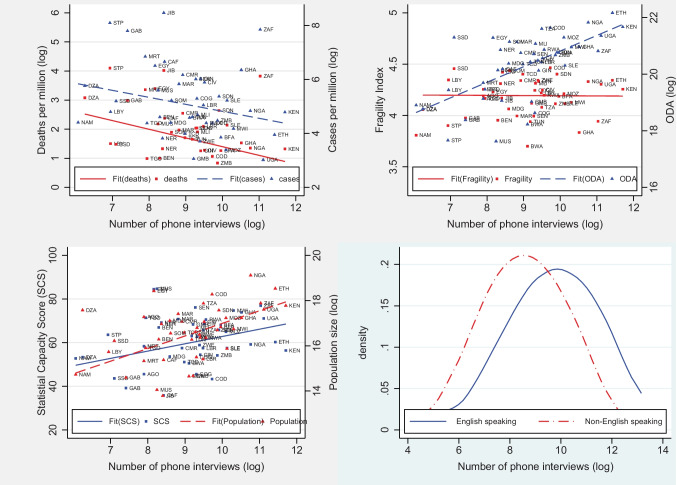
Correlates of phone survey intensity in Africa. Notes: The fit line is from a linear regression of the number of interviews (log) on population (log), statistical capacity score (SCS), net official development assistance (ODA) (log), fragile state index (FSI), and number of COVID related cases/deaths. Source: Computed from data compiled by the authors

Generally, one might expect differences in resources or COVID cases and deaths to drive survey locations. However, a pairwise correlation result presented in Table 8 in the appendix indicates that this is not the case (also see Fig. 3, top left panel)^9^. Instead, survey location choices are related to the size of the country (population size) and the availability of pre-existing data or indeed statistical capacity (SCS) (bottom left panel). Figure 3 also shows that phone surveys are less (more) correlated with fragility index (net official development assistance (ODA) (top right panel). Finally, the bottom right panel shows that phone surveys are more prevalent in countries with English as an official language, which is consistent with other studies (Porteous, 2020).

The use of representative and up-to-date pre-pandemic baseline data as a sampling frame in phone surveys is vital to correct the biases associated with the sample selection process (Ambel et al., 2021). In line with this, 39% of all phone surveys and 73% of panel surveys in our review used pre-pandemic face-to-face survey datasets as a baseline. When used, the preferred baseline data are large-scale, representative, long-running, and integrated panel surveys (Panel B, Table 2). However, since the pre-pandemic distribution of large-scale datasets across African countries is highly uneven (Porteous, 2020), this has led to a significant disparity in data collected during the pandemic as shown above.

(D) Geospatial coding

One of the downsides of using a phone survey, compared to alternative ways of data collection, is that it allows for limited sets of questions to be included. Fortunately, there is large useful and open access information (e.g. diseases statistics, government measures, public goods, price trends, conflicts, weather data, etc.) that can be extracted and spatially and temporally matched with survey data. To take advantage of this, a few phone surveys include either location information or use baseline data that already collected GPS information. However, this is not widespread. In our review, only 39% of the surveys include such information (Table 3).

(E) Open access of the data

Another desirable, yet largely missing, quality of phone surveys is the availability of the resulting data as open access for public use. While this enables widespread use of the data, it would also allow pooling across surveys in cross-country analyses. Regardless, only 24% of the phone surveys are currently available for public use (Table 2). Eight (~ 3.42%) other surveys are not yet open access but indicated that the corresponding data will become open access in the future. For the remaining others, we are unable to find information to determine if they will become open access or not. The most popular of the open access data is from the World Bank data portal, based on which the Bank creates harmonized indicators and disseminates through High-Frequency Monitoring Dashboard.^10^

To summarize and further elaborate on the above five dimensions, we generated an index representing the pooling potential of the reviewed phone surveys based on 14 selected survey and questionnaire features, each of which is coded as a binary variable that takes a value of 1 if desirable, 0 otherwise. The index, thus, ranges from zero to 14. The selected 14 survey and questionnaire features include, (i) survey is panel; (ii) survey involves continuous data collection; (iii) survey data available for public use; (iv) survey sample is large (greater than 1000 respondents); (v) questionnaire includes standardized food security questions; (vi) questionnaire include change in employment; (vii) questionnaire includes change in income; (viii) questionnaire includes access to services; (ix) questionnaire includes mental health questions; (x) questionnaire includes coping mechanisms; (xi) survey allows merging with external data; (xii) survey uses pre-crisis baseline data; (xiii) pre-crisis baseline is representative and (xiv) survey covers both urban and rural areas.

**Fig. 4:**
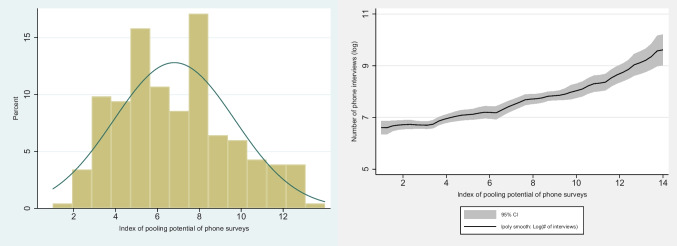
Pooling potential of phone survey (right) and correlation with survey intensity in Africa (right). Source: Computed from data compiled by the authors

In our study, this indicator takes an average overall value of 6.8 (out of the possible 14 points), suggesting that many studies do not fulfill the requirements which would allow pooling across surveys (Fig. 4). Furthermore, the right-hand side graph indicates that this quality of phone surveys is positively correlated with the number of interviews suggesting that less studied countries are disadvantaged not only in terms of survey intensity but also in terms of pooling potential to study cross-regional and cross-country issues.

## Discussion

Our review of phone surveys in Africa during the COVID-19 pandemic provides several interesting insights. First, we find that the distribution of phone surveys in the continent is highly uneven - and systematically so. The variation across countries is strongly related to factors that are broad indicators of the perceived costs or ease of research (population size, inflow of aid, statistical capacity, and fragility of economies) rather than the potential benefits to the communities (e.g. Coronavirus incidence). Since the distribution of data was already uneven before the pandemic, and that design of quality phone surveys benefits from the availability of representative and up-to-date pre-pandemic data (Ambel et al., 2021), COVID-19 may have perpetuated (or even exacerbated) the existing uneven distribution of data between countries in Africa. Since the uneven distribution of data implies an uneven distribution of research (Brück et al., 2014), which in turn translates into an uneven evidence base for policy-makers (Porteous, 2020), we call on the scientific community to focus further research on locations where the evidence base is thin. Policymakers in less researched areas could also help attract more research by improving their statistical capacity, openness, and governance.

Our review indicates that the current intensity of data collection is strongly influenced by past scores of statistical capacity. Indeed, countries that were able to rapidly launch phone surveys at the beginning of the pandemic were those with long-term and representative pre-pandemic data that serves as a sampling frame as well as with up-to-date information and communication technology (ICT) infrastructure for the implementation of longitudinal household surveys (Gourlay et al., 2021; UNDESA and World Bank, 2020). This suggests that countries should invest in ICT infrastructure, particularly on National Statistical Offices (NSOs) to provide them with reliable internet access and computer hardware and software for data collection, storage, and processing.

Second, our results demonstrate that the existing surveys mostly focus on a narrow theme and only some of them allow for heterogeneous analysis across socioeconomic, spatial, and intertemporal dimensions. Most surveys focus on food security (even if not measured consistently), employment, and income losses. Non-economic aspects such as the interaction with or the impact of the pandemic on mental health, social capital, trust, governance, and intra-household relationships are not fully accommodated. As these are important correlates of household welfare and are significantly affected by the pandemic (Brooks et al., 2020; Ravens-Sieberer et al., 2021), their exclusion from surveys could lead to an underestimation of the impact of the pandemic.

Third, most of the surveys were designed as short-term projects. While it has so far been natural to focus on the pandemic’s short-term impact, it is also critical to monitor how the pandemic unfolds and assess its implications for medium- and long-term food security to inform policy decisions. For instance, the large-scale countermeasures implemented in most countries have changed patterns in education attendance, consumption, and household labor allocation (IFPRI, 2020). While the short-term effects of these changes are profound, they are also likely to determine the speed of recovery and the long-term growth trajectory of affected households and countries. Furthermore, it is not yet clear whether and how these COVID-shaped trajectories may interact with existing vulnerabilities such as old age, household size, income sources, or poverty.

Fourth, only 24% of the phone surveys are available for public use. This constraints widespread distribution and use of the data to support research-based policy solutions. Even when the data are available, the idiosyncratic nature of many surveys prevents meaningful pooling of surveys across Africa, closing an avenue of learning open to standardized surveys like DHS, LSMS, or MICS. Furthermore, it is relatively less common for researchers and statistical offices in Africa to register their surveys and projects in international registries. This reduces the potential synergies among different projects from harmonization of survey instruments. A widespread registry of surveys also helps to identify and draw attention to relatively understudied areas and topics.

Finally, due to a lack of geospatial information or alternative location information, most surveys are not suitable for matching with secondary sources of information on, inter alia, diseases statistics, government measures, price trends, conflicts, or weather data, reducing the scope for multidisciplinary research around the pandemic.

To address some of the shortcomings in the extant surveys, we designed the Life with Corona - Africa (LwC-Africa) survey. LwC-Africa is based in four African countries — Uganda, Tanzania, Sierra Leone, and Mozambique and builds on and complements the global LwC online survey (https://lifewithcorona.org/). The survey is based on country representative samples and allows statistically meaningful and valid comparisons between and within countries across different socio-demographic groups (e.g., age, gender, and place of residence). The survey follows a stratified random sampling method and interviewed 500 respondents per month per country over 12 months in 2021. The questionnaire contains modules on COVID exposure and experiences on a wide range of topics, including economic, health, social, psychological, and political issues. Specifically, the questionnaire contains the following six modules: (i) household demographic characteristics; (ii) Coronavirus exposure; (iii) Economic well-being, financial insecurity, coping mechanisms, and external support; (iv) Social capital; (v) Food and nutrition security; and (vi) Mental health and wellbeing. It also allows geospatial matching with secondary data sources. We will avail the data for research and public use upon publication of this article.

## Conclusion

The COVID-19 pandemic is a global crisis with multiple interlinked dimensions, including health, economic, social, and political consequences. Yet, the effects differ significantly across and within countries, over time, and among individuals based on sociodemographic characteristics and place of residence. Therefore, in order to clearly understand the evolution and the socio-economic impacts of and responses to the pandemic, surveys would benefit from collecting data across multiple countries, multiple topics, continuously throughout the pandemic and allow matching with external datasets, such as disease statistics or information on countermeasures.

However, our review indicates that phone surveys in Africa are concentrated in a few countries; mostly focusing on a narrow theme and a single country; and only a few allow heterogeneous analyses across socioeconomic, spatial, and intertemporal dimensions. We, therefore, highlight the importance for the scientific community to focus its research much more on countries (and regions and groups within countries) as well as topics where the evidence base is thin. Longer-term studies with more continuous data collection would help understand the complex dynamics that COVID-19 will have for food security specifically and societies in general in Africa. More geo-coding and more standardized study protocols would allow creation of synergies between surveys, akin to large-scale data programs like DHS, LSMS, and MICS. Policymakers can also attract more research on food security in less researched areas by improving their statistical capacity, openness, and governance.

## Appendix

**Table 5:** Major lead organizations of the survey

Leading organizations	Frequency	Survey rounds	Sample size	Survey duration*
Multinational agencies^a^	71	3.0	1,000	12
Research & survey consultants^b^	50	2.0	632	6
CGIAR Research Centers^c^	12	1.5	522	5
Consortiums^d^	51	2.0	1,057	6
Universities^e^	46	1.0	1,004	4
Country statistical offices	4	2.5	2,180	9
Total	234	2	1,001	6

^a^This includes the World Bank, the World Food Program (WFP), European commission, CDC Africa, and ActionAid

^b^This includes 60 Decibels, BRAC international, GeoPoll, IDInsight, Innovation for Poverty Action (IPA) and FinMark Trust

^c^This includes International Food Policy Research Institute (IFPRI), International Livestock Research Institute (ILRI) and The International Center for Agriculture Research in the Dry Areas (ICARDA)

^d^This includes Partnership for Evidence-based COVID-19 Response (PERC), Future Agricultures Consortium (FAC), and Leibniz Institute for Vegetable and Ornamental Crops (IGZ)/ International Security and Development Center (ISDC)

^e^This includes international universities (e.g. UC Berkeley, MIT, University of Oxford)

*Survey duration is measured in months

**Table 6:** Description of Life with Corona Africa (LwC-Africa) Survey

LwC-Africa builds on and complements the global LwC online survey (https://lifewithcorona.org/). The survey is based on country representative samples and allows statistically meaningful and valid comparisons between and within countries across different socio-demographic groups (e.g., age, gender, and place of residence). The survey follows a stratified random sampling method to interview 500 respondents per month per country over 12 months. The questionnaire contains modules on COVID exposure and experiences on a wide range of topics, including economic, health, social, psychological, and political issues. Specifically, the questionnaire contains the following six modules: (i) household demographic characteristics; (ii) Coronavirus exposure; (iii) Economic well-being, financial insecurity, coping mechanisms, and external support; (iv) Social capital; (v) Food and nutrition security; and (vi) Mental health and wellbeing. It also allows geospatial matching with secondary data sources.

**Table 7:** Key demographic, economic, and Covid-19 related indicators in Africa

Variable	Mean	Median	Std.Dev
Population size, mln.	24.6	12.8	35.1
GDP per capita, USD	5,994	3,489	6,394
Net Official Development Assistance received (% of GNI)	7.5	5.3	9.9
Share of households with electricity access (%)	54.2	50.3	26.7
Mobile cellular subscriptions (per 100 people), 2018	84.6	82.4	38.7
Mobile cellular subscriptions (per 100 people), 2019	86.4	86.1	38.9
Mobile cellular subscriptions (per 100 people), 2020	87.9	91.5	38.6
Statistical Capacity Score	58.7	59.7	13.8
Fragility Index	85.0	86.0	15.1
Total COVID cases per million, 2020	2,502	960	4,194
Total COVID cases per million, 2021	9,927	2,404	24,322
Total COVID deaths per million, 2020	43.3	14.4	80.1
Total COVID deaths per million, 2021	146.1	37.7	261.1

The indicators are for the year 2020 unless indicated otherwise

Sources: World Development Indicators, World Bank (2022); Fund for Peace (FFP), and ourworldindata.org

**Table 8:** Pairwise correlation between survey intensity indicators and sociodemographic characteristics in Africa

	COVID cases per million	COVID deaths per million	GDP per capita	Access to electricity	Mobile subscription	Statistical Capacity Score	Fragility Index	Population	ODA	# surveys	# surveys rounds	# respondents	# interviews
COVID cases per million	1												
COVID deaths per million	0.90	1											
GDP per capita	0.20	0.24	1										
Access to electricity	0.40	0.42	0.72	1									
Mobile subscription	0.07	0.09	0.63	0.71	1								
Statistical Capacity Score	0.01	0.13	-0.06	0.19	0.31	1							
Fragility Index	-0.22	-0.21	-0.68	-0.58	-0.64	-0.41	1						
Population size	-0.14	-0.06	-0.06	0.08	-0.06	0.15	0.22	1					
ODA	-0.27	-0.27	-0.34	-0.18	-0.25	0.13	0.38	0.76	1				
# surveys	-0.17	-0.23	-0.22	0.02	0.07	0.23	0.05	0.42	0.62	1			
# surveys rounds	-0.20	-0.26	-0.28	-0.09	-0.02	0.21	0.13	0.50	0.70	0.96	1		
# respondents	-0.13	-0.16	-0.17	0.03	0.09	0.16	0.07	0.40	0.56	0.97	0.95	1	
# interviews	-0.08	-0.09	-0.15	0.03	0.05	0.18	0.09	0.49	0.67	0.87	0.93	0.90	1

Sources: data on COVID caseloads and deaths are extracted from ourworldindata.org; data on GDP, ODA, population size, access to electricity, and mobile subscription are from World Development Indicators, World Bank (2022); Fragility index is from Fund for Peace (FFP); total and average number of ongoing phone is computed from data compiled by the authors

**Table 9:** Complete list of phone surveys on COVID-19 and food security in Africa

No	Survey Description	Lead organization	Start time	country	Sample size	Food Security included?	Food security measures				Date updated	Internet link
							Access to food?	FIES?	Food gap?	FCS?		
1	Household Monitoring Survey (HMS)	The World Bank	6/1/2020	Central African Republic	600	yes	yes	yes	yes	no	30-Sep-21	www.worldbank.org
2	Household Monitoring Survey (HMS)	The World Bank	8/1/2020	Central African Republic	1266	yes	yes	yes	yes	no	1-Nov-21	www.worldbank.org
3	Household Monitoring Survey (HMS)	The World Bank	5/1/2020	Gabon	1656	yes	yes	yes	yes	no	28-Sep-21	www.worldbank.org
4	High Frequency Phone Survey (HFPS)	The World Bank	5/1/2020	Chad	1748	yes	yes	yes	yes	no	29-Sep-21	https://microdata.worldbank.org
5	High Frequency Phone Survey (HFPS)	The World Bank	8/1/2020	Gambia	1437	yes	yes	yes	yes	no	30-Sep-21	www.worldbank.org
6	Household Monitoring Survey (HMS)	The World Bank	6/1/2020	Liberia	1920	yes	yes	yes	yes	no	1-Nov-21	www.worldbank.org
7	High Frequency Phone Survey (HFPS)	The World Bank	5/1/2020	Mauritius	924	yes	yes	yes	no	no	1-Nov-21	www.worldbank.org
8	High Frequency Phone Survey (HFPS)	The World Bank	5/1/2020	Tunisia	1032	yes	yes	yes	yes	no	17-Oct-21	www.worldbank.org
9	High Frequency Phone Survey (HFPS)	The world Bank, LSMS	4/1/2020	Ethiopia	3200	yes	yes	yes	yes	no	30-Sep-21	www.worldbank.org
10	High Frequency Phone Survey (HFPS)	The world Bank, LSMS	4/1/2020	Nigeria	1950	yes	yes	yes	yes	no	16-Feb-20	www.worldbank.org
11	High Frequency Phone Survey (HFPS)	The world Bank, LSMS	4/1/2020	Malawi	2337	yes	yes	yes	yes	no	16-Feb-20	www.worldbank.org
12	High Frequency Phone Survey (HFPS)	The world Bank, LSMS	6/1/2020	Uganda	2226	yes	yes	yes	yes	no	31-Aug-21	https://microdata.worldbank.org
13	High Frequency Phone Survey (HFPS)	The World Bank	6/1/2020	Mali	1700	yes	yes	yes	yes	no	1-Nov-21	www.worldbank.org
14	High Frequency Phone Survey (HFPS)	The World Bank	7/1/2020	Djibouti	1486	yes	yes	yes	yes	no	1-Nov-21	https://microdata.worldbank.org
15	Household Monitoring Survey (HMS)	The World Bank	6/1/2020	South Sudan	1213	yes	yes	yes	yes	no	2-Mar-21	www.worldbank.org
16	Household Monitoring Survey (HMS)	The World Bank	6/1/2020	Zambia	1602	yes	yes	yes	yes	no	18-Oct-21	www.worldbank.org
17	High Frequency Phone Survey (HFPS)	The world Bank	6/1/2020	Zimbabwe	1747	yes	yes	yes	yes	no	March 30 2021	www.worldbank.org
18	High Frequency Phone Survey (HFPS)	The world Bank	6/1/2020	Congo, Dem. Rep.	1082	yes	yes	yes	yes	no	30-Sep-21	www.worldbank.org
19	Household Monitoring Survey (HMS)	The World Bank	6/1/2020	Madagascar	1240	yes	yes	yes	yes	no	18-Oct-21	www.worldbank.org
20	Household Monitoring Survey (HMS)	The World Bank	5/1/2020	Senegal	1220	yes	yes	yes	yes	no	17-Oct-21	www.worldbank.org
21	High Frequency Phone Survey (HFPS)	The World Bank	5/1/2020	Kenya	5389	yes	yes	yes	yes	no	8-Mar-21	https://microdata.worldbank.org
22	Household Monitoring Survey (HMS)	The World Bank	6/1/2020	Ghana	3265	yes	yes	yes	yes	no	1-Nov-21	www.worldbank.org
23	High Frequency Phone Survey (HFPS)	The World Bank	7/1/2020	Guinea	1968	yes	yes	yes	yes	no	1-Oct-21	www.worldbank.org
24	Hunger and COVID 19 tracking survey	World Food Program (WFP)	4/1/2020	Libya	521	yes	yes	no	yes	yes	1-Nov-21	https://hungermap.wfp.org
25	High Frequency Phone Survey (HFPS)	The World Bank	6/1/2020	Mozambique	1097	yes	yes	yes	yes	no	18-Oct-21	www.worldbank.org
26	High Frequency Phone Survey (HFPS)	The World Bank	11/1/2020	Rwanda	1396	yes	yes	yes	yes	no	1-Nov-21	www.worldbank.org
27	High Frequency Phone Survey (HFPS)	The World Bank	6/1/2020	Sudan	4032	yes	yes	yes	yes	no	18-Oct-21	www.worldbank.org
28	Household Monitoring Survey (HMS)	The World Bank	7/1/2020	Sierra Leone	6570	yes	yes	yes	yes	no	31-Mar-21	www.worldbank.org
29	Household Monitoring Survey (HMS)	The World Bank	7/1/2020	Somalia	2811	yes	yes	yes	yes	no	1-Nov-21	www.worldbank.org
30	Household Monitoring Survey (HMS)	The World Bank	7/1/2020	Sao Tome and Principe	1025	yes	yes	yes	yes	no	18-Oct-21	https://microdata.worldbank.org
31	High Frequency Phone Survey (HFPS)	The World Bank	9/1/2020	Congo, Rep.	1386	yes	yes	yes	yes	no	30-Sep-21	www.worldbank.org
32	PERC COVID-19 survey 1	PERC	3/1/2020	Cameroon	1043	yes	yes	no	no	no	01. Oct 21	https://www.ipsos.com
33	PERC COVID-19 survey 2	PERC	8/1/2020	Cameroon	1449	yes	yes	no	yes	no	01. Oct 21	https://www.ipsos.com
34	PERC COVID-19 survey 1	PERC	4/1/2020	Cote d’Ivoire	1036	yes	yes	no	no	no	01. Oct 21	https://www.ipsos.com
35	PERC COVID-19 survey 2	PERC	8/1/2020	Cote d’Ivoire	1416	yes	yes	no	yes	no	01. Oct 21	https://www.ipsos.com
36	PERC COVID-19 survey 1	PERC	4/1/2020	Congo, Dem. Rep.	1009	yes	yes	no	no	no	01. Oct 21	https://www.ipsos.com
37	PERC COVID-19 survey 2	PERC	8/1/2020	Congo, Dem. Rep.	1351	yes	yes	no	yes	no	01. Oct 21	https://www.ipsos.com
38	PERC COVID-19 survey 1	PERC	3/1/2020	Egypt, Arab Rep.	1098	yes	yes	no	no	no	01. Oct 21	https://www.ipsos.com
39	PERC COVID-19 survey 2	PERC	8/1/2020	Egypt, Arab Rep.	1206	yes	yes	no	yes	no	01. Oct 21	https://www.ipsos.com
40	PERC COVID-19 survey 1	PERC	3/1/2020	Ethiopia	1021	yes	yes	no	no	no	01. Oct 21	https://www.ipsos.com
41	PERC COVID-19 survey 2	PERC	8/1/2020	Ethiopia	1571	yes	yes	no	yes	no	01. Oct 21	https://www.ipsos.com
42	PERC COVID-19 survey 1	PERC	3/1/2020	Ghana	1001	yes	yes	no	no	no	01. Oct 21	https://www.ipsos.com
43	PERC COVID-19 survey 2	PERC	8/1/2020	Ghana	1338	yes	yes	no	yes	no	01. Oct 21	https://www.ipsos.com
44	PERC COVID-19 survey 1	PERC	4/1/2020	Guinea	1034	yes	yes	no	no	no	01. Oct 21	https://www.ipsos.com
45	PERC COVID-19 survey 2	PERC	8/1/2020	Guinea	1283	yes	yes	no	yes	no	01. Oct 21	https://www.ipsos.com
46	PERC COVID-19 survey 1	PERC	3/1/2020	Kenya	1031	yes	yes	no	no	no	01. Oct 21	https://www.ipsos.com
47	PERC COVID-19 survey 2	PERC	8/1/2020	Kenya	1224	yes	yes	no	yes	no	01. Oct 21	https://www.ipsos.com
48	PERC COVID-19 survey 1	PERC	4/1/2020	Liberia	1059	yes	yes	no	no	no	01. Oct 21	https://www.ipsos.com
49	PERC COVID-19 survey 2	PERC	8/1/2020	Liberia	1366	yes	yes	no	yes	no	01. Oct 21	https://www.ipsos.com
50	PERC COVID-19 survey 1	PERC	4/1/2020	Morocco	1045	yes	yes	no	no	no	01. Oct 21	https://www.ipsos.com
51	PERC COVID-19 survey 1	PERC	3/1/2020	Mozambique	1057	yes	yes	no	no	no	01. Oct 21	https://www.ipsos.com
52	PERC COVID-19 survey 2	PERC	8/1/2020	Mozambique	1314	yes	yes	no	yes	no	01. Oct 21	https://www.ipsos.com
53	PERC COVID-19 survey 1	PERC	3/1/2020	Nigeria	1068	yes	yes	no	no	no	01. Oct 21	https://www.ipsos.com
54	PERC COVID-19 survey 2	PERC	8/1/2020	Nigeria	1304	yes	yes	no	yes	no	01. Oct 21	https://www.ipsos.com
55	PERC COVID-19 survey 1	PERC	4/1/2020	Senegal	1039	yes	yes	no	no	no	01. Oct 21	https://www.ipsos.com
56	PERC COVID-19 survey 2	PERC	8/1/2020	Senegal	1259	yes	yes	no	yes	no	01. Oct 21	https://www.ipsos.com
57	PERC COVID-19 survey 1	PERC	4/1/2020	South Africa	1099	yes	yes	no	no	no	01. Oct 21	https://www.ipsos.com
58	PERC COVID-19 survey 2	PERC	8/1/2020	South Africa	1395	yes	yes	no	yes	no	01. Oct 21	https://www.ipsos.com
59	PERC COVID-19 survey 1	PERC	3/1/2020	Sudan	1101	yes	yes	no	no	no	01. Oct 21	https://www.ipsos.com
60	PERC COVID-19 survey 2	PERC	8/1/2020	Sudan	1438	yes	yes	no	yes	no	01. Oct 21	https://www.ipsos.com
61	PERC COVID-19 survey 1	PERC	3/1/2020	Tanzania	1103	yes	yes	no	no	no	01. Oct 21	https://www.ipsos.com
62	PERC COVID-19 survey 1	PERC	4/1/2020	Tunisia	1004	yes	yes	no	no	no	01. Oct 21	https://www.ipsos.com
63	PERC COVID-19 survey 2	PERC	8/1/2020	Tunisia	1218	yes	yes	no	yes	no	01. Oct 21	https://www.ipsos.com
64	PERC COVID-19 survey 1	PERC	3/1/2020	Uganda	1073	yes	yes	no	no	no	01. Oct 21	https://www.ipsos.com
65	PERC COVID-19 survey 2	PERC	8/1/2020	Uganda	1286	yes	yes	no	yes	no	01. Oct 21	https://www.ipsos.com
66	PERC COVID-19 survey 1	PERC	3/1/2020	Zambia	1035	yes	yes	no	no	no	01. Oct 21	https://www.ipsos.com
67	PERC COVID-19 survey 2	PERC	8/1/2020	Zambia	1290	yes	yes	no	yes	no	01. Oct 21	https://www.ipsos.com
68	PERC COVID-19 survey 1	PERC	4/1/2020	Zimbabwe	1034	yes	yes	no	no	no	01. Oct 21	https://www.ipsos.com
69	PERC COVID-19 survey 2	PERC	8/1/2020	Zimbabwe	1333	yes	yes	no	yes	no	01. Oct 21	https://www.ipsos.com
70	Rural household phone survey	IFPRI	6/1/2020	Senegal	750	yes	yes	yes	yes	yes	1-Nov-21	https://dataverse.harvard.edu
71	Rural household phone survey	IFPRI	8/1/2020	Ghana	543	yes	yes	yes	yes	yes	2-Nov-21	https://dataverse.harvard.edu
72	Rural household phone survey	IFPRI	8/1/2020	Malawi	1020	yes	yes	yes	yes	yes	2-Mar-21	https://ebrary.ifpri.org/digital
73	Rural household Phone survey	IFPRI	10/1/2020	Niger	403	yes	yes	yes	yes	yes	1-Nov-21	https://dataverse.harvard.edu
74	Rural household phone survey	IFPRI	8/1/2020	Nigeria	1000	yes	yes	yes	yes	yes	1-Mar-21	https://www.ifpri.org/
75	Phone survey of PSNP households	IFPRI	6/1/2020	Ethiopia	1500	yes	yes	yes	yes	yes	16-Feb-21	https://www.ifpri.org/
76	Lagos Trader Survey	Stanford University	4/1/2020	Nigeria	765	yes	no	no	no	no	16-Feb-21	https://www.poverty-action.org/
77	Life with Corona Africa survey	IGZ and ISDC	1/1/2021	Uganda	500	yes	yes	yes	yes	yes	30-Sep-21	https://www.igzev.de
78	Life with Corona Africa survey	IGZ and ISDC	1/1/2021	Tanzania	500	yes	yes	yes	yes	yes	30-Sep-21	https://www.igzev.de
79	Life with Corona Africa survey	IGZ and ISDC	1/1/2021	Sierra Leone	500	yes	yes	yes	yes	yes	30-Sep-21	https://www.igzev.de
80	Life with Corona Africa survey	IGZ and ISDC	1/1/2021	Mozambique	500	yes	yes	yes	yes	yes	30-Sep-21	https://www.igzev.de
81	Smallholder Farmer Perceptions about COVID-19	Kansas State University	6/1/2020	Senegal	872	yes	yes	no	no	no	19-Mar-21	https://www.sciencedirect.com
82	Survey of weekly Financial and Health Diaries	Vrije Universiteit & Tinbergen Institute	2/1/2020	Kenya	328	yes	yes	no	yes	no	16-Feb-21	https://www.aighd.org
83	Survey on COVID-19 Knowledge and Social Distancing	University of Massachusetts	4/1/2020	Ghana	362	yes	yes	no	no	no	16-Feb-21	https://www.poverty-action.org
84	Survey on COVID-19 Knowledge and Social Distancing	University of Massachusetts	4/1/2020	Malawi	563	yes	yes	no	no	no	16-Feb-21	https://www.poverty-action.org
85	Survey on COVID-19 Knowledge and Social Distancing	University of Massachusetts	4/1/2020	Sierra Leone	633	yes	yes	no	no	no	16-Feb-21	https://www.poverty-action.org
86	Survey on COVID-19 Knowledge and Social Distancing	University of Massachusetts	4/1/2020	Tanzania	557	yes	yes	no	no	no	16-Feb-21	https://www.poverty-action.org
87	Survey on delivery of health & nutrition services	IFPRI	12/1/2020	Ethiopia	233	yes	yes	no	no	no	1-Nov-21	https://www.poverty-action.org
88	Survey on Economic Outcomes and Resilience to COVID-19	UC Berkeley	4/27/2020	Kenya	1500	yes	yes	yes	yes	yes	1-Nov-21	https://kenyacovidtracker.org
89	Survey on impact of Covid-19 on economic outcomes	University of Oxford	8/1/2020	Uganda	1250	yes	yes	no	yes	no	1-Nov-21	https://www.poverty-action.org
90	Survey on knowledge and perceptions of coronavirus	GeoPoll	4/1/2020	Benin	400	yes	yes	no	no	no	16-Feb-21	https://www.geopoll.com
91	Survey on knowledge and perceptions of coronavirus	GeoPoll	4/1/2020	Congo, Dem. Rep.	400	yes	yes	no	no	no	16-Feb-21	https://www.geopoll.com
92	Survey on knowledge and perceptions of coronavirus	GeoPoll	4/1/2020	Ghana	400	yes	yes	no	no	no	16-Feb-21	https://www.geopoll.com
93	Survey on knowledge and perceptions of coronavirus	GeoPoll	4/1/2020	Cote d’Ivoire	400	yes	yes	no	no	no	16-Feb-21	https://www.geopoll.com
94	Survey on knowledge and perceptions of coronavirus	GeoPoll	4/1/2020	Kenya	400	yes	yes	no	no	no	16-Feb-21	https://www.geopoll.com
95	Survey on knowledge and perceptions of coronavirus	GeoPoll	4/1/2020	Mozambique	400	yes	yes	no	no	no	16-Feb-21	https://www.geopoll.com
96	Survey on knowledge and perceptions of coronavirus	GeoPoll	4/1/2020	Nigeria	400	yes	yes	no	no	no	16-Feb-21	https://www.geopoll.com
97	Survey on knowledge and perceptions of coronavirus	GeoPoll	4/1/2020	Rwanda	400	yes	yes	no	no	no	16-Feb-21	https://www.geopoll.com
98	Survey on knowledge and perceptions of coronavirus	GeoPoll	4/1/2020	South Africa	400	yes	yes	no	no	no	16-Feb-21	https://www.geopoll.com
99	Survey on knowledge and perceptions of coronavirus	GeoPoll	4/1/2020	Uganda	400	yes	yes	no	no	no	16-Feb-21	https://www.geopoll.com
100	Survey on knowledge and perceptions of coronavirus	GeoPoll	4/1/2020	Zambia	400	yes	yes	no	no	no	16-Feb-21	https://www.geopoll.com
101	Survey on knowledge and perceptions of coronavirus	GeoPoll	4/1/2020	Tanzania	400	yes	yes	no	no	no	16-Feb-21	https://www.geopoll.com
102	Survey on the effect of COVID-19 Across the Globe	BRAC International	4/1/2020	Tanzania	241	yes	yes	no	yes	no	16-Feb-21	http://www.brac.net
103	Survey on the effect of COVID-19 Across the Globe	BRAC International	4/1/2020	Uganda	352	yes	yes	no	yes	no	16-Feb-21	http://www.brac.net
104	Survey on the effect of COVID-19 Across the Globe	BRAC International	4/1/2020	Sierra Leone	219	yes	yes	no	yes	no	16-Feb-21	http://www.brac.net
105	Survey on the effect of COVID-19 Across the Globe	BRAC International	4/1/2020	Liberia	223	yes	yes	no	yes	no	16-Feb-21	http://www.brac.net
106	Survey on the effect of COVID-19 Across the Globe	BRAC International	4/1/2020	Rwanda	151	yes	yes	no	yes	no	16-Feb-21	http://www.brac.net
107	Effect of the pandemic in poor urban neighborhoods	ETH Zurich	5/1/2020	Ghana	993	yes	yes	no	yes	no	12-Feb-21	https://dec.ethz.ch
108	Effect of the pandemic in poor urban neighborhoods	ETH Zurich	5/1/2020	South Africa	385	yes	yes	no	yes	no	12-Feb-21	https://dec.ethz.ch
109	Survey on the Impact of COVID-19 and Cash Transfers	University of California	5/1/2020	Liberia	593	yes	yes	yes	no	no	16-Feb-21	https://www.poverty-action.org
110	Survey on the Impact of COVID-19 and Cash Transfers	University of California	5/1/2020	Malawi	596	yes	yes	yes	no	no	16-Feb-21	https://www.poverty-action.org
111	COVID-19 on Low-Income Customers of Social Enterprises	60 Decibels	4/1/2020	Cote d’Ivoire	768	yes	yes	no	no	no	19-Oct-21	https://app.60decibels.com
112	COVID-19 on Low-Income Customers of Social Enterprises	60 Decibels	4/1/2020	Congo, Dem. Rep.	398	yes	yes	no	no	no	19-Oct-21	https://app.60decibels.com
113	COVID-19 on Low-Income Customers of Social Enterprises	60 Decibels	4/1/2020	Ghana	419	yes	yes	no	no	no	19-Oct-21	https://app.60decibels.com
114	COVID-19 on Low-Income Customers of Social Enterprises	60 Decibels	4/1/2020	Kenya	2398	yes	yes	no	no	no	19-Oct-21	https://app.60decibels.com
115	COVID-19 on Low-Income Customers of Social Enterprises	60 Decibels	4/1/2020	Madagascar	210	yes	yes	no	no	no	19-Oct-21	https://app.60decibels.com
116	COVID-19 on Low-Income Customers of Social Enterprises	60 Decibels	4/1/2020	Nigeria	3432	yes	yes	no	no	no	19-Oct-21	https://app.60decibels.com
117	COVID-19 on Low-Income Customers of Social Enterprises	60 Decibels	4/1/2020	Rwanda	1084	yes	yes	no	no	no	19-Oct-21	https://app.60decibels.com
118	COVID-19 on Low-Income Customers of Social Enterprises	60 Decibels	4/1/2020	Senegal	252	yes	yes	no	no	no	19-Oct-21	https://app.60decibels.com
119	COVID-19 on Low-Income Customers of Social Enterprises	60 Decibels	4/1/2020	Sierra Leone	1529	yes	yes	no	no	no	19-Oct-21	https://app.60decibels.com
120	COVID-19 on Low-Income Customers of Social Enterprises	60 Decibels	4/1/2020	South Africa	1009	yes	yes	no	no	no	19-Oct-21	https://app.60decibels.com
121	COVID-19 on Low-Income Customers of Social Enterprises	60 Decibels	4/1/2020	Tanzania	2663	yes	yes	no	no	no	19-Oct-21	https://app.60decibels.com
122	COVID-19 on Low-Income Customers of Social Enterprises	60 Decibels	4/1/2020	Uganda	2652	yes	yes	no	no	no	19-Oct-21	https://app.60decibels.com
123	COVID-19 on Low-Income Customers of Social Enterprises	60 Decibels	4/1/2020	Zambia	790	yes	yes	no	no	no	19-Oct-21	https://app.60decibels.com
124	Survey on the impacts of COVID-19 on Pastoralist communities	ILRI	8/1/2020	Kenya	100	yes	yes	no	yes	no	16-Feb-21	https://www.poverty-action.org
125	Survey on the value of ICT during COVID-19	Georgia State Universisty	9/1/2020	Ghana	2019	yes	yes	no	no	no	2-Mar-21	https://economicsobservatory.com
126	Covid-19 on the distribution of youth-led businesses	University of Exeter	12/1/2020	Kenya	1000	yes	yes	no	no	no	16-Feb-21	https://www.poverty-action.org
127	WB Household Monitoring Survey (HMS)	The World Bank	5/1/2020	Togo	2189	yes	yes	yes	yes	no	21-Nov-22	https://www.worldbank.org
128	The RECOVR survey	Innovation for poverty action (IPA)	6/1/2020	Burkina Faso	1356	yes	yes	no	yes	no	16-Feb-21	https://www.poverty-action.org
129	The RECOVR survey	Innovation for poverty action (IPA)	6/1/2020	Cote d’Ivoire	1329	yes	yes	no	yes	no	16-Feb-21	https://www.poverty-action.org
130	The RECOVR survey	Innovation for poverty action (IPA)	5/1/2020	Ghana	1633	yes	yes	no	yes	no	16-Feb-21	https://www.poverty-action.org
131	The RECOVR survey	Innovation for poverty action (IPA)	6/1/2020	Rwanda	1482	yes	yes	no	yes	no	16-Feb-21	https://www.poverty-action.org
132	The RECOVR survey	Innovation for poverty action (IPA)	5/1/2020	Sierra Leone	1304	yes	yes	no	yes	no	16-Feb-21	https://www.poverty-action.org
133	The RECOVR survey	Innovation for poverty action (IPA)	5/1/2020	Uganda	1250	yes	yes	no	yes	no	16-Feb-21	https://www.poverty-action.org
134	The RECOVR survey	Innovation for poverty action (IPA)	6/1/2020	Zambia	1278	yes	yes	no	yes	no	16-Feb-21	https://www.poverty-action.org
135	Vegetable value chain survey	IFPRI	5/1/2020	Ethiopia	433	yes	yes	no	yes	yes	16-Feb-21	http://essp.ifpri.info
136	Hunger and COVID 19 tracking survey	World Food Program (WFP)	4/1/2020	Angola	896	yes	yes	no	yes	yes	1-Nov-21	https://hungermap.wfp.org
137	Hunger and COVID 19 tracking survey	World Food Program (WFP)	4/1/2020	Benin	896	yes	yes	no	yes	yes	1-Nov-21	https://hungermap.wfp.org
138	Hunger and COVID 19 tracking survey	World Food Program (WFP)	4/1/2020	Burkina Faso	896	yes	yes	no	yes	yes	1-Nov-21	https://hungermap.wfp.org
139	Hunger and COVID 19 tracking survey	World Food Program (WFP)	4/1/2020	Cameroon	896	yes	yes	no	yes	yes	1-Nov-21	https://hungermap.wfp.org
140	Hunger and COVID 19 tracking survey	World Food Program (WFP)	4/1/2020	Central African Republic	896	yes	yes	no	yes	yes	1-Nov-21	https://hungermap.wfp.org
141	Hunger and COVID 19 tracking survey	World Food Program (WFP)	4/1/2020	Chad	896	yes	yes	no	yes	yes	1-Nov-21	https://hungermap.wfp.org
142	Hunger and COVID 19 tracking survey	World Food Program (WFP)	4/1/2020	Congo, Dem. Rep.	896	yes	yes	no	yes	yes	1-Nov-21	https://hungermap.wfp.org
143	Hunger and COVID 19 tracking survey	World Food Program (WFP)	4/1/2020	Congo, Rep.	896	yes	yes	no	yes	yes	1-Nov-21	https://hungermap.wfp.org
144	Hunger and COVID 19 tracking survey	World Food Program (WFP)	4/1/2020	Cote d’Ivoire	896	yes	yes	no	yes	yes	1-Nov-21	https://hungermap.wfp.org
145	Hunger and COVID 19 tracking survey	World Food Program (WFP)	4/1/2020	Ethiopia	896	yes	yes	no	yes	yes	1-Nov-21	https://hungermap.wfp.org
146	Hunger and COVID 19 tracking survey	World Food Program (WFP)	4/1/2020	Guinea	896	yes	yes	no	yes	yes	1-Nov-21	https://hungermap.wfp.org
147	Hunger and COVID 19 tracking survey	World Food Program (WFP)	4/1/2020	Kenya	896	yes	yes	no	yes	yes	1-Nov-21	https://hungermap.wfp.org
148	Hunger and COVID 19 tracking survey	World Food Program (WFP)	4/1/2020	Liberia	896	yes	yes	no	yes	yes	1-Nov-21	https://hungermap.wfp.org
149	Hunger and COVID 19 tracking survey	World Food Program (WFP)	4/1/2020	Madagascar	896	yes	yes	no	yes	yes	1-Nov-21	https://hungermap.wfp.org
150	Hunger and COVID 19 tracking survey	World Food Program (WFP)	4/1/2020	Malawi	896	yes	yes	no	yes	yes	1-Nov-21	https://hungermap.wfp.org
151	Hunger and COVID 19 tracking survey	World Food Program (WFP)	4/1/2020	Mali	896	yes	yes	no	yes	yes	1-Nov-21	https://hungermap.wfp.org
152	Hunger and COVID 19 tracking survey	World Food Program (WFP)	4/1/2020	Mauritania	896	yes	yes	no	yes	yes	1-Nov-21	https://hungermap.wfp.org
153	Hunger and COVID 19 tracking survey	World Food Program (WFP)	4/1/2020	Mozambique	896	yes	yes	no	yes	yes	1-Nov-21	https://hungermap.wfp.org
154	Hunger and COVID 19 tracking survey	World Food Program (WFP)	4/1/2020	Niger	896	yes	yes	no	yes	yes	1-Nov-21	https://hungermap.wfp.org
155	Hunger and COVID 19 tracking survey	World Food Program (WFP)	4/1/2020	Nigeria	896	yes	yes	no	yes	yes	1-Nov-21	https://hungermap.wfp.org
156	Hunger and COVID 19 tracking survey	World Food Program (WFP)	4/1/2020	Sierra Leone	896	yes	yes	no	yes	yes	1-Nov-21	https://hungermap.wfp.org
157	Hunger and COVID 19 tracking survey	World Food Program (WFP)	4/1/2020	Somalia	896	yes	yes	no	yes	yes	1-Nov-21	https://hungermap.wfp.org
158	COVID-19 follow-up phone survey	Uni-Bonn-ILR	6/1/2020	Namibia	430	yes	yes	no	no	no	21-Nov	http://www.ilr.uni-bonn.de
159	Hunger and COVID 19 tracking survey	World Food Program (WFP)	4/1/2020	Tanzania	896	yes	yes	no	yes	yes	1-Nov-21	https://hungermap.wfp.org
160	Hunger and COVID 19 tracking survey	World Food Program (WFP)	4/1/2020	Zambia	896	yes	yes	no	yes	yes	1-Nov-21	https://hungermap.wfp.org
161	Hunger and COVID 19 tracking survey	World Food Program (WFP)	4/1/2020	Zimbabwe	896	yes	yes	no	yes	yes	1-Nov-21	https://hungermap.wfp.org
162	Young Lives phone survey Young	University of Oxford, Young Lives	6/1/2020	Ethiopia	2500	yes	yes	no	yes	no	1-Nov-21	https://beta.ukdataservice.ac.uk
163	Changes in Norms about Social Distancing	University of Michigan & IPA	7/1/2020	Mozambique	2415	yes	yes	no	yes	no	1-Nov-21	https://www.poverty-action.org
164	Addis Ababa Covid19 phone survey	IFPRI	5/1/2020	Ethiopia	600	yes	yes	yes	yes	yes	01. Oct 21	https://economicsobservatory.com
165	APRA COVID-19 Rapid Assessment	APRA, Future Agricultures Consortium (FAC)	6/1/2020	Ethiopia	107	yes	yes	yes	no	no	01. Oct 21	https://opendocs.ids.ac.uk
166	APRA COVID-19 Rapid Assessment	APRA, Future Agricultures Consortium (FAC)	6/1/2020	Ghana	110	yes	yes	yes	no	no	01. Oct 21	https://opendocs.ids.ac.uk
167	APRA COVID-19 Rapid Assessment	APRA, Future Agricultures Consortium (FAC)	6/1/2020	Kenya	100	yes	yes	yes	no	no	01. Oct 21	https://opendocs.ids.ac.uk
168	APRA COVID-19 Rapid Assessment	APRA, Future Agricultures Consortium (FAC)	6/1/2020	Malawi	114	yes	yes	yes	no	no	01. Oct 21	https://opendocs.ids.ac.uk
169	APRA COVID-19 Rapid Assessment	APRA, Future Agricultures Consortium (FAC)	6/1/2020	Nigeria	111	yes	yes	yes	no	no	01. Oct 21	https://opendocs.ids.ac.uk
170	APRA COVID-19 Rapid Assessment	APRA, Future Agricultures Consortium (FAC)	6/1/2020	Tanzania	102	yes	yes	yes	no	no	01. Oct 21	https://opendocs.ids.ac.uk
171	APRA COVID-19 Rapid Assessment	APRA, Future Agricultures Consortium (FAC)	6/1/2020	Zimbabwe	107	yes	yes	yes	no	no	01. Oct 21	https://opendocs.ids.ac.uk
172	APRA COVID-19 Rapid Assessment	APRA, Future Agricultures Consortium (FAC)	10/1/2020	Zambia	115	yes	yes	yes	no	no	01. Oct 21	https://opendocs.ids.ac.uk
173	Cash transfers and COVID-19	IDInsight	7/1/2020	Uganda	633	yes	yes	yes	yes	no	4-Oct-21	https://www.idinsight.org
174	Coronavirus Rapid Mobile Survey (CRAM)	University of Stellenbosch, UCT & Wits	5/1/2020	South Africa	10,000	yes	yes	yes	yes	yes	4-Oct-21	https://cramsurvey.org/
175	COVID-19 and food insecurity in Cameroon	University of Douala, Cameroon.	3/1/2020	Cameroon	487	yes	yes	no	no	no	04. Oct 2021	https://gsconlinepress.com
176	WB High Frequency Phone Survey (HFPS)	The world Bank	4/1/2020	Ethiopia	3896	yes	yes	no	yes	no	04. Oct 21	https://osf.io/wvf7m/
177	WB High Frequency Phone Survey (HFPS)	The world Bank	6/1/2020	Burkina Faso	1968	yes	yes	yes	yes	no	30-Sep-21	www.worldbank.org
178	COVID-19 Livelihoods Impact Tracking Survey	FinMark Trust & insight2impact facility	4/1/2020	Ghana	1000	yes	yes	no	yes	no	March 20,2021	https://covid19tracker.africa/
179	COVID-19 Livelihoods Impact Tracking Survey	FinMark Trust & insight2impact facility	4/1/2020	Kenya	1000	yes	yes	no	yes	no	March 20,2021	https://covid19tracker.africa/
180	COVID-19 Livelihoods Impact Tracking Survey	FinMark Trust & insight2impact facility	4/1/2020	Nigeria	1800	yes	yes	no	yes	no	March 20,2021	https://covid19tracker.africa/
181	COVID-19 Livelihoods Impact Tracking Survey	FinMark Trust & insight2impact facility	4/1/2020	Rwanda	1000	yes	yes	no	yes	no	March 20,2021	https://covid19tracker.africa/
182	COVID-19 Livelihoods Impact Tracking Survey	FinMark Trust & insight2impact facility	4/1/2020	South Africa	1000	yes	yes	no	yes	no	March 20,2021	https://covid19tracker.africa/
183	COVID-19 Livelihoods Impact Tracking Survey	FinMark Trust & insight2impact facility	4/1/2020	Uganda	1200	yes	yes	no	yes	no	March 20,2021	https://covid19tracker.africa/
184	COVID-19 Livelihoods Impact Tracking Survey	FinMark Trust & insight2impact facility	4/1/2020	Zambia	1000	yes	yes	no	yes	no	March 20,2021	https://covid19tracker.africa/
185	COVID-19 Phone Survey in Senegal	Center for Global Development (CGD)	4/1/2020	Senegal	1023	yes	yes	no	yes	no	1-Nov-21	http://crdes.sn/
186	Escaping Poverty—COVID-19 Phone Survey	Northwestern University	9/1/2020	Ghana	7330	yes	yes	no	no	no	16-Feb-21	https://www.poverty-action.org
187	WB Household Monitoring Survey (HMS)	The World Bank	4/1/2020	Cote d’Ivoire	800	yes	yes	yes	yes	no	21-Nov	https://worldbank.org
188	Impacts of the COVID-19 crises on rural household	European Commission JRC	5/1/2020	Cote d’Ivoire	1547	yes	yes	no	no	no	21-Nov	
189	Survey on knowledge and behaviors related to COVID-19	NA	4/1/2020	Malawi	619	yes	yes	no	no	no	March 31 2021	https://demographic-research.org
190	The effect of COVID on households	NA	4/1/2020	Morocco	2350	yes	yes	yes	yes	yes	March 31 2021	https://www.hcp.ma
191	Survey of households and frims in Kenya	NA	4/1/2020	Kenya	2000	yes	yes	no	yes	no	21-Nov	https://socialscienceregistry.org
192	Survey on the impact of cash in a crisis	NA	4/1/2020	Kenya	800	yes	yes	no	no	no	21-Nov	https://economicsobservatory.com
193	Survey on the impact of cash in a crisis	NA	4/1/2020	Uganda	600	yes	yes	no	yes	no	21-Nov	https://pedl.cepr.org/
194	community-led WASH Survey	NA	6/1/2020	Congo, Dem. Rep.	1328	yes	yes	no	yes	no	21-Nov	https://poverty-action.org/
195	Survey of Low-Income Customers of Social Enterprises	60 Decibels	4/1/2020	Benin	515	yes	yes	no	no	no	19-Oct-21	https://app.60decibels.com
196	Survey of Low-Income Customers of Social Enterprises	60 Decibels	4/1/2020	Cameroon	366	yes	yes	no	no	no	19-Oct-21	https://app.60decibels.com
197	Rapid Multi-Country Survey on the Impact of the COVID-19	PARG	4/1/2020	Kenya	973	yes	yes	no	yes	no	21-Nov	https://precisiondev.org
198	Secondary Education and Coping with a Pandemic	NA	5/1/2020	Ghana	682	yes	yes	no	no	no	21-Nov	https://www.poverty-action.org
199	Survey on Cash and Compliance with Social Distancing	NA	6/1/2020	Ghana	1500	yes	yes	no	yes	no	21-Nov	https://www.poverty-action.org
200	COVID-19, Gender, and Youth Employment in Kenya	NA	12/1/2020	Kenya	2000	yes	no	no	yes	no	21-Nov	https://socialscienceregistry.org
201	Digital Credit Usage & Intra-household Bargaining Patterns	NA	4/1/2021	Kenya	7836	yes	yes	no	no	no	21-Nov	https://socialscienceregistry.org
202	Parent-Child Preferences and Secondary School Choice	UC Berkele	3/1/2020	Kenya	2973	yes	yes	no	no	no	21-Nov	https://www.poverty-action.org
203	Resilience to economic shocks through continued electricity access	UC Berkele	12/1/2020	Kenya	2000	yes	yes	yes	no	no	21-Nov	https://socialscienceregistry.org
204	Understanding Effects and Resilience	UC Berkele	4/1/2020	Kenya	1008	yes	yes	yes	no	no	21-Nov	https://www.poverty-action.org
205	The Effects of a Universal Basic Income	MIT	6/1/2020	Kenya	8605	yes	yes	yes	yes	no	21-Nov	https://www.poverty-action.org
206	Cash Transfers During a Pandemic	Arizona State University	4/1/2020	Kenya	753	yes	yes	no	yes	no	21-Nov	https://www.poverty-action.org
207	Kenya rapid phone-based surveys	Population counsil	3/1/2020	Kenya	2009	yes	no	no	yes	no	21-Nov	https://dataverse.harvard.edu
208	Kenya rapid phone-based surveys	Population counsil	7/1/2020	Kenya	6853	yes	no	no	yes	no	21-Nov	https://dataverse.harvard.edu
209	Resilience & Risk in the Informal Sector	Stanford University	5/1/2020	Nigeria	868	yes	yes	no	yes	no	21-Nov	https://www.poverty-action.org
210	Survey of Low-Income Customers of Social Enterprises	60 Decibels	4/1/2020	Togo	630	yes	yes	no	no	no	19-Oct-21	https://app.60decibels.com
211	Entrepreneurship Education in Uganda	UC Berkeley & Educate	9/1/2020	Uganda	1916	yes	yes	no	no	no	21-Nov	https://socialscienceregistry.org
212	COVID-19 Shock on the youth	Center for Effective Global Action (CEGA)	5/1/2020	Uganda	1920	yes	yes	no	yes	no	21-Nov	https://poverty-action.org
213	Collective Action of School Leaders during Coronavirus Pandemic	Innovation for poverty action (IPA) & Elevate	5/1/2020	Uganda	88	no	no	no	no	no	21-Nov	https://poverty-action.org
214	Community Health Care and COVID-19 Pandemic	Trinity College Dublin & Stockholm University	6/1/2020	Uganda	2000	yes	yes	no	yes	no	21-Nov	https://socialscienceregistry.org
215	Vulnerability and Trust in the Aftermath of COVID-19	Columbia University	6/1/2020	Uganda	2700	yes	yes	yes	yes	no	21-Nov	https://wzb-ipi.github.io
216	Public Health, Trust, and Livelihoods	Brown University	7/1/2020	Uganda	2587	yes	yes	no	yes	no	21-Nov	https://cega.berkeley.edu
217	Randomized Impact Evaluation	Northwestern University & IPA		Uganda	6631	yes	yes	yes	yes	no	21-Nov	https://socialscienceregistry.org
218	Consumer Protection Survey	Innovation for poverty action (IPA)	5/1/2020	Uganda	830	yes	yes	no	no	no	21-Nov	https://dataverse.harvard.edu
219	Peer Messaging to Reduce Covid-19 Transmission	NA	6/1/2020	Zambia	2000	yes	yes	no	yes	no	21-Nov	https://socialscienceregistry.org
220	Household survey and COVID-19 follow-up phone survey	Uni-Bonn-ILR	6/1/2020	Kenya	654	yes	yes	no	no	no	21-Nov	http://www.ilr.uni-bonn.de
221	Household survey and COVID-19 follow-up phone survey	Uni-Bonn-ILR	6/1/2020	Tanzania	680	yes	yes	no	no	no	21-Nov	http://www.ilr.uni-bonn.de
222	Rural household phone survey	IFPRI	8/1/2020	Nigeria	501	yes	yes	yes	yes	yes	21-Nov	https://dataverse.harvard.edu
223	Phone farm survey about the impact of COVID-19	ICARDA	5/1/2020	Tunisia	100	yes	yes	no	yes	no	21-Nov	https://data.mel.cgiar.org
224	Stemming Learning Loss During the Pandemic	Columbia University & Young love	5/1/2020	Botswana	4500	no	no	no	no	no	21-Nov	https://socialscienceregistry.org
225	COVID-19 Household Impact Survey	World Food Program (WFP)	5/1/2020	Algeria	517	yes	yes	yes	yes	no	21-Nov	https://docs.wfp.org
226	CDC-Africa public opionion survey	CDC Africa	10/1/2020	Ethiopia	1001	yes	yes	no	no	no	21-Nov	https://africacdc.org
227	CDC-Africa public opionion survey	CDC Africa	11/1/2020	Kenya	1000	yes	yes	no	no	no	21-Nov	https://africacdc.org
228	CDC-Africa public opionion survey	CDC Africa	9/1/2020	Morocco	1000	yes	yes	no	no	no	21-Nov	https://africacdc.org
229	CDC-Africa public opionion survey	CDC Africa	9/1/2020	Tunisia	1000	yes	yes	no	no	no	21-Nov	https://africacdc.org
230	Fever survey	Mauritious statistical office	4/1/2020	Mauritius	1042	no	no	no	no	no	21-Nov	https://reliefweb.int
231	COVID-19 on poverty and living standards	University of Cape Coast	6/1/2020	Ghana	900	yes	yes	yes	yes	yes	21-Nov	https://www.tandfonline.com
232	IMPACT OF COVID-19 ON YOUNG WOMEN	ActionAid	8/1/2020	Ghana	305	yes	yes	no	no	no	21-Nov	https://actionaid.org
233	IMPACT OF COVID-19 ON YOUNG WOMEN	ActionAid	8/1/2020	Kenya	200	yes	yes	no	no	no	21-Nov	https://actionaid.org
234	IMPACT OF COVID-19 ON YOUNG WOMEN	ActionAid	8/1/2020	South Africa	300	yes	yes	no	no	no	21-Nov	https://actionaid.org

## Footnotes

1 The UNDESA report indicates that in May 2020, 97% of NSOs in sub-Saharan African countries were not able to meet international reporting requirements due to the pandemic, as opposed to 38.5% in NSOs in high-income countries.

2 Phone surveys generally take three forms. First type is Computer Assisted Telephone Interviews (CATI), where interview responses are recorded on a computer based on an interactive survey questionnaire. Second type is Interactive Voice Response (IVR) where data is collected based on automated, prerecorded questions. The third category is text message-based surveys (SMS) where questions are sent and responses are collected using text messages. Most COVID-19 monitoring surveys in Africa used CATI mode which, compared to the IVR and SMS modes, is more expensive but allows potential depth and breadth of data gathering (Glazerman et al., 2020).

3 Our review includes only household and individual level surveys. A major excluded survey is firm/company level surveys. All African countries are included in the search. However, survey descriptions from eight countries - Burundi, Cabo Verde, Comoros, Eritrea, Eswastini (formerly named Swaziland), Equatorial Guinea, Guinea Bissau, and Lesotho - is not included in the paper because we didn’t find any phone survey information from these countries.

4 The bank maintains a “COVID-19 High-Frequency Monitoring Dashboard” to trace and update surveys and to generate and regularly disseminate harmonized indicators for public consumption. The dashboard is accessible at http://bit.ly/wbcovid19dashboard.

5 Details on the methodology including the list of surveyed countries, daily and weekly COVID-19 and hunger snapshots are accessible at https://hungermap.wfp.org/.

6 The other three surveys are related to learning loss during the pandemic, country’s preparedness and response capacities to the pandemic and collective action of school leaders during the pandemic.

7 See https://datatopics.worldbank.org/statisticalcapacity/.

8 See https://fragilestatesindex.org/.

9 Table 7 in the Appendix presents a descriptive statistics of key demographic, economic, and COVID-19 related indicators across the countries in the continent. The result shows notable differences among the included countries. However, these differences don’t seem to derive the choices of survey locations (See Table 8 in the Appendix).

10 The dashboard is accessible through: http://bit.ly/wbcovid19dashboard.

## References

[CR1] Abate, G. T., Brauw, A., De, Hirvonen, K., & Wolle, A. (2021). Measuring Consumption over the Phone: Evidence from a Survey Experiment in Urban Ethiopia (IFPRI Discussion Paper 02087, Issue December). 10.1016/j.jdeveco.2022.103026 PMC9711906 36471688

[CR2] Abreu, L., Koebach, A., Díaz, O., Carleial, S., Hoeffler, A., Stojetz, W., Freudenreich, H., Justino, P., & Brück, T. (2021). Life With Corona: Increased Gender Differences in Aggression and Depression Symptoms Due to the COVID-19 Pandemic Burden in Germany. 10.3389/fpsyg.2021.689396 10.3389/fpsyg.2021.689396 PMC8353131 34385959

[CR3] Ambel, A., McGee, K., & Tsegay, A. (2021). Reducing Bias in Phone Survey Samples Effectiveness of Reweighting Techniques Using Face-to-Face Surveys as Frames in Four African Countries (Policy Research Working Paper 9676; Issue May).

[CR4] Ambler K, Herskowitz S, Maredia MK (2021). Are we done yet? Response fatigue and rural livelihoods. Journal of Development Economics.

[CR5] Brooks SK, Webster RK, Smith LE, Woodland L, Wessely S, Greenberg N, Rubin GJ (2020). The psychological impact of quarantine and how to reduce it: rapid review of the evidence. The Lancet.

[CR6] Brück T, Esenaliev D, Kroeger A, Kudebayeva A, Mirkasimov B, Steiner S (2014). Household survey data for research on well-being and behavior in Central Asia. Journal of Comparative Economics.

[CR7] Brülhart M, Klotzbücher V, Lalive R, Reich SK (2021). Mental health concerns during the COVID-19 pandemic as revealed by helpline calls. Nature.

[CR8] Cafiero C, Viviani S, Nord M (2018). Food security measurement in a global context: The food insecurity experience scale. Measurement.

[CR9] Dabalen, A., Etang, A., Hoogeveen, J., Mushi, E., Schipper, Y., & von Engelhardt, J. (2016). Mobile Phone Panel Surveys in Developing Countries: A Practical Guide for Microdata Collection. The World Bank. 10.1596/978-1-4648-0904-0

[CR10] Das J, Do QT, Shaines K, Srikant S (2013). U.S. and them: The geography of academic research. Journal of Development Economics.

[CR11] Dasgupta S, Robinson EJZ (2022). Impact of COVID-19 on food insecurity using multiple waves of high frequency household surveys. Scientific Reports.

[CR12] Delius, A., Himelein, K., & Pape, U. J. (2020). Conducting Rapid Response Phone Surveys (RRPS) to Fill Data Gaps. Poverty and Equity Notes. https://openknowledge.worldbank.org/handle/10986/34300

[CR13] Dillon B (2012). Using mobile phones to collect panel data in developing countries. Journal of International Development.

[CR14] Djankov, S., & Panizza, U. (Eds.). (2020). COVID-19 in Developing Economies. Centre for Economic Policy Research.

[CR15] Egger, D., Miguel, E., Warren, S. S., Shenoy, A., Collins, E., Karlan, D., Parkerson, D., Mobarak, A. M., Fink, G., Udry, C., Walker, M., Haushofer, J., Larreboure, M., Lopez-Pena, S. A., Benhachmi, P., Humphreys, S., Lowe, M., Meriggi, L., Wabwire, N. F., Davis, A., & Vernot, C. A. (2021). C. Falling Living Standards during the COVID-19 Crisis: Quantitative Evidence from Nine Developing Countries. *Science Advances*, *April*. 10.1126/sciadv.abe0997 PMC7864564 33547077

[CR16] Glazerman, S., Rosenbaum, M., Sandino, R., & Shaughnessy, L. (2020). Remote Surveying in a Pandemic: Handbook Executive Summary. https://www.poverty-action.org/publication/remote-surveying-pandemic-handbook

[CR17] Gourlay S, Kilic T, Martuscelli A, Wollburg P, Zezza A (2021). Viewpoint: High-frequency phone surveys on COVID-19: Good practices, open questions. Food Policy.

[CR18] Hirvonen K, de Brauw A, Abate GT (2021). Food Consumption and Food Security during the COVID-19 Pandemic in Addis Ababa. American Journal of Agricultural Economics.

[CR19] Hoogeveen, J., & Pape, U. (Eds.). (2020). Data collection in fragile states: Innovations from Africa and beyond. Palgrave Macmillan. 10.1007/978-3-030-25120-8

[CR20] IFPRI. (2020). In J. Swinnen, & J. Mcdermott (Eds.), Covid19 & Global Food Security. International Food Policy Research Institute (IFPRI).

[CR21] Jaacks LM, Veluguri D, Serupally R, Roy A, Prabhakaran P, Ramanjaneyulu G (2021). Impact of the COVID-19 pandemic on agricultural production, livelihoods, and food security in India: baseline results of a phone survey. Food Security.

[CR22] Kühne, S., Kroh, M., Liebig, S., & Zinn, S. (2020). The Need for Household Panel Surveys in Times of Crisis: The Case of SOEP-CoV. *Survey Research Methods*.

[CR23] Laborde D, Martin W, Vos R (2021). Impacts of COVID-19 on global poverty, food security, and diets: Insights from global model scenario analysis. Agricultural Economics.

[CR24] Nechifor V, Ramos MP, Ferrari E, Laichena J, Kihiu E, Omanyo D, Musamali R, Kiriga B (2021). Food security and welfare changes under COVID-19 in Sub-Saharan Africa: Impacts and responses in Kenya. Global Food Security.

[CR25] Porteous, O. (2020). Research Deserts and Oases: Evidence from 27 Thousand Economics Journal Articles on Africa (Working Paper).

[CR26] Poudel D, Gopinath M (2021). Exploring the disparity in global food security indicators. Global Food Security.

[CR27] Ravens-Sieberer, U., Kaman, A., Erhart, · Michael, Devine, J., Schlack, R., & Otto, C. (2021). Impact of the COVID-19 pandemic on quality of life and mental health in children and adolescents in Germany. *European Child & Adolescent Psychiatry*, *1*, 3. 10.1007/s00787-021-01726-5 10.1007/s00787-021-01726-5 PMC7829493 33492480

[CR28] Robinson MD, Hartley JE, Schneider PH (2006). Which countries are studied most by economists? An examination of the regional distribution of economic research. Kyklos.

[CR29] Stojetz W, Ferguson NTN, Baliki G, Botía OD, Elfes J, Esenaliev D, Freudenreich H, Koebach A, de Lopes L, Peitz L, Todua A, Schreiner M, Hoeffler A, Justino P, Brück T (2022). The life with corona survey. Social Science & Medicine.

[CR30] Sturges JE, Hanrahan KJ (2004). Comparing telephone and face-to-face qualitative interviewing: a research note. Qualitative Research.

[CR31] UNDESA and World Bank. (2020). Monitoring the state of statistical operations under the COVID-19 Pandemic Highlights from a global COVID-19 survey of National Statistical Offices. Issue August).

[CR32] World Bank (2009). World Development Report: reshaping economic geography. The world Bank.

[CR33] World Bank (2022). World development indicators. https://databank.worldbank.org/source/world-development-indicators

